# Breast-conserving surgery is not associated with increased local recurrence in patients with early-stage node-negative triple-negative breast cancer treated with neoadjuvant chemotherapy

**DOI:** 10.1016/j.breast.2024.103701

**Published:** 2024-02-24

**Authors:** David Krug, Valentina Vladimirova, Michael Untch, Thorsten Kühn, Andreas Schneeweiss, Carsten Denkert, Beyhan Ataseven, Christine Solbach, Bernd Gerber, Hans Tesch, Michael Golatta, Sabine Seiler, Jörg Heil, Valentina Nekljudova, Johannes Holtschmidt, Sibylle Loibl

**Affiliations:** a Department of Radiation Oncology, University Hospital Schleswig-Holstein, Kiel, Germany; b German Breast Group, Neu-Isenburg, Germany; c HELIOS Klinikum Berlin-Buch, Berlin, Germany; d Department of Gynecology and Obstetrics, University of Ulm, Ulm, Germany; e National Center for Tumor Diseases, University Hospital and German Cancer Research Center, Heidelberg, Germany; f Institut für Pathologie, Philipps-Universität Marburg und Universitätsklinikum Marburg (UKGM), Marburg, Germany; g Department of Gynecology, Gynecologic Oncology and Obstetrics, Bielefeld University, Medical School and University Medical Center OWL, Klinikum Lippe, Detmold, Germany; h Goethe University Frankfurt, Department of Gynecology and Obstetrics, University Hospital, Germany; i Department of Obstetrics and Gynecology, University of Rostock, Rostock, Germany; j Center for Hematology and Oncology Bethanien, Frankfurt, Germany; k Department of Gynecology and Obstetrics, University of Heidelberg, Heidelberg, Germany; l Brustzentrum Heidelberg Klinik St. Elisabeth, Max-Reger-Straße 5-7, 69121 Heidelberg, Germany; m Department of Gynecology and Obstectrics, Die Filderklinik, Filderstadt, Germany

**Keywords:** Breast-conserving surgery, Local recurrence, Primary systemic therapy, Mastectomy, Molecular subtype, Previous presentation

## Abstract

**Background:**

Neoadjuvant chemotherapy (NACT) is routinely used for patients with triple-negative breast cancer (TNBC). Upfront breast-conserving therapy (BCT) consisting of breast-conserving surgery (BCS) and adjuvant radiotherapy (RT) has been shown to be associated with improved outcome in patients with early TNBC as compared to mastectomy.

**Methods:**

We identified 2632 patients with early TNBC from the German Breast Group meta-database. Patients with cT1-2 cN0 and ypN0, available surgery and follow-up data were enrolled. Data of 1074 patients from 8 prospective NACT trials were available. Endpoints of interest were locoregional recurrence as first site of relapse (LRR), disease-free survival (DFS) and overall survival (OS). We performed univariate and multivariate Fine-Gray analysis and Cox regression models.

**Results:**

After a median follow-up of 64 months, there were 94 (8.8%) locoregional events as first site of relapse. Absence of pathologic complete response (pCR) was associated with increased LRR upon uni- and multivariate analysis (hazard ratio [HR] = 2.28; p < 0.001 and HR = 2.22; p = 0.001). Type of surgery was not associated with LRR. Patients in the BCS-group had better DFS and OS (DFS: HR = 0.47; p < 0.001 and OS: HR = 0.40; p < 0.001). BCS was associated with improved DFS and OS upon multivariate analysis (DFS: HR = 0.51; p < 0.001; and OS HR = 0.43; p < 0.001), whereas absence of pCR was associated with worse DFS and OS (DFS: HR = 2.43; p < 0.001; and OS: HR = 3.15; p < 0.001).

**Conclusions:**

In this retrospective analysis of patients with early stage node-negative TNBC treated with NACT, BCS was not associated with an increased risk of LRR but with superior DFS and OS.

## Highlights

• This is an analysis of 1074 patients with early triple-negative breast cancer.
• Breast conservation was not associated with increased locoregional recurrence.
• Improved overall and disease-free survival was noted with breast conservation.
• Patients with pathologic complete response had superior outcome.

## 1 Introduction

Breast-conserving therapy (BCT) consisting of breast-conserving surgery (BCS) followed by adjuvant radiotherapy (RT) is the standard of care regarding operative management of the primary tumor for patients with early-stage breast cancer [1]. Adjuvant RT is routinely used after BCS and is associated with a 15.7% absolute and 52% relative risk reduction for any recurrence at 10 years and an 3.8% improvement in breast cancer-related mortality at 15 years as shown by the Early Breast Cancer Trialists’ Collaborative Group meta-analysis (EBCTCG) [1]. Population-based analysis from the Netherlands and Sweden have suggested improved survival in patients treated with BCS and adjuvant RT (breast-conserving therapy = BCT) [2,3].

Triple-negative breast cancer (TNBC) is characterized by limited pharmaceutical treatment options as therapy relevant expression of estrogen receptor (ER), progesterone receptor (PR) and human epidermal growth factor receptor-2 (HER2) is missing. Hence, TNBC is associated with an inferior prognosis compared to other breast cancer subtypes [4]. Previous work has also demonstrated elevated rates of locoregional recurrence (LRR) in patients with TNBC [[5], [6], [7], [8]]. Retrospective data suggested that BCT significantly decreases the risk of LRR in patients with early stage TNBC compared to mastectomy [9].

Neoadjuvant chemotherapy (NACT) is the standard of care for patients with early TNBC [10]. The prognostic value of pathologic complete response (pCR) is well described [11] and the option to escalate post-neoadjuvant treatment is subject of past and ongoing trials [[12], [13], [14]]. Multi-agent chemotherapy results in pCR-rates of >50% in patients with TNBC [15,16]. Recently, the addition of checkpoint inhibitors to NACT demonstrated improvements in pCR-rates and survival outcomes [[17], [18], [19], [20]]. Post-neoadjuvant treatment with Capecitabine or Olaparib has been shown to significantly improve overall survival (OS) in patients with TNBC with residual invasive disease after NACT [21,22]. Data on LRR stratified by biological subtype in patients that underwent NACT are limited. Two retrospective reports demonstrated an association of TNBC with increased risk of LRR while pCR was associated with improved outcome [23,24].

An EBCTCG-metaanalysis on chemotherapy sequence demonstrated comparable survival outcome with adjuvant chemotherapy compared to NACT [25]. However, the risk of LRR was significantly increased in patients treated with NACT. This was most evident in trials that used radiotherapy without surgery in patients with a clinical complete remission but was also present in patients treated with BCT. Furthermore, the increase in LRR was more pronounced in patients that were downstaged from a planned mastectomy to BCT. However, only one single trial that used anthracycline/taxane containing NACT vs the same regimen given adjuvant was included. Moreover, it is to be questioned whether the lack of availability for modern preoperative imaging techniques and refined resection margin evaluation had an impact on surgical accuracy as the included trials recruited from 1983 until 2002.

The lack of data with more contemporary management and according to subtypes prompted us to investigate whether the choice of local therapy has an impact on the prognosis of patients with early TNBC treated with NACT.

### 1.1 Patients and methods

The GBG meta-database of neoadjuvant trials was queried for patients with TNBC. A total of 2632 patients from 8 prospective trials were identified. These trials were GeparDuo, GeparTrio, GeparQuattro, GeparQuinto, GeparSixto, GeparSepto and GeparOcto. All these trials were focused on systemic therapy improvement in the neoadjuvant setting. Study protocols contained recommendations for locoregional management, including surgery of the breast and axilla and adjuvant radiotherapy.

**Fig. 1:**
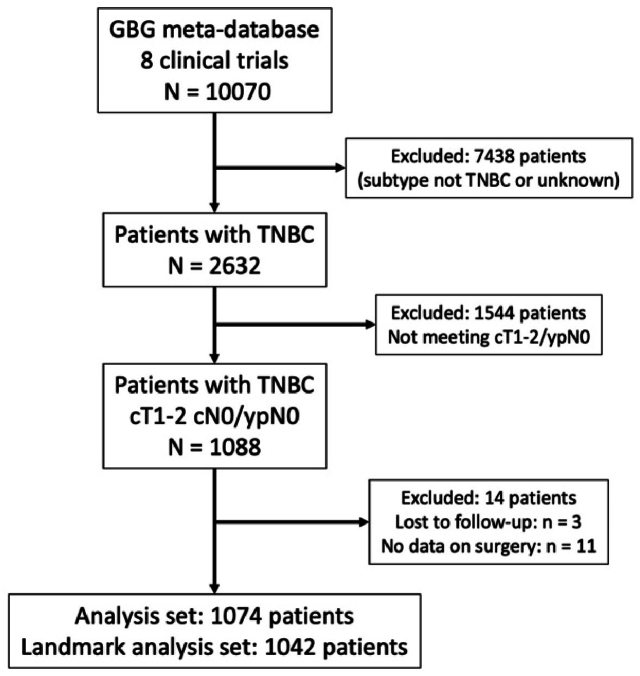
Patient flowchart for this analysis. GBG = German Breast Group; TNBC = triple negative breast cancer.

For the purpose of this project, patients with early-stage TNBC defined as cT1-2 cN0 with pathologically negative lymph nodes after NACT (ypN0), available surgery and follow-up data were considered eligible. Fig. 1 shows the flowchart for patient inclusion. After exclusion of ineligible patients, 1074 patients from 8 prospective GBG-trials were included. All included trials were approved by the responsible ethics committees at the participating institutions. All patients provided their informed consent before enrolment in the respective trials.

LRR as first event (other sites of recurrence were considered competing events), disease-free survival (DFS) and OS were defined as endpoints of interest and reported using cumulative incidence (for LRR [26]) and Kaplan-Meier estimates (for DFS and OS). Patient characteristics were described according to type of surgery. The Pearson's chi-squared test (for categorical parameters with more than two categories) and Fisher's exact test (for binary parameters) were used to assess the comparability between groups. Hazard ratios (HRs) with 95% confidence interval (CI) were assessed in univariate and multivariate analyses using Fine-Gray's (for LRR) and Cox (for DFS and OS) regression models. Multivariate regression models were adjusted for covariables study, age, cT, surgery type and pCR. For the analyses including pCR as covariable, only patients at risk at the landmark time were evaluated. Data on adjuvant RT were not available in the GBG meta-database. Hence, we will use the term BCS to describe locoregional management, although the overwhelming majority of patient most likely underwent adjuvant radiotherapy. In a previous analysis of three prospective GBG-trials, 98.5% of patients received adjuvant RT after BCS [27,28]. Furthermore, we selected a subgroup of patients that did not have a recommendation for adjuvant post-mastectomy RT in the respective study protocols. pCR was defined as the absence of any residual invasive tumor (ypT0/is ypN0). All statistical tests were two-sided, with values of p < 0.05 considered statistically significant. All statistical analyses were performed using SPSS 22.0 (IBM SPSS Statistics 22) and SAS (version 9.4).

## 2 Results

Patients were enrolled between June 1999 and June 2016 and patient characteristics are shown in Table 1. The median follow-up was 64.4 (range 3–224.8) months. Type of surgery was BCS for 916 patients (85.3%) and mastectomy for 158 patients (14.7%). Patients who underwent BCS were older regarding the whole population (age ≤50 years 59.9% and age >50 years 40.1% vs. age ≤50 years 72.8% and age >50 years 27.2%; p = 0.002) and more frequently displayed a pCR (60.0%) in comparison to patients underwent mastectomy (55.2%, p < 0.001) while there were no significant differences regarding clinical tumor stage, grading and histologic type. BRCA-1/2 mutation was significantly more common among patients that underwent mastectomy (39.4% vs. 13.9%; p < 0.001).

**Table 1:** Patient characteristics.

**Parameter**	**Category**	BCS (N = 916)	Mastectomy (N = 158)	Total (N = 1074)	*P*-value (BCS vs. M)
		N (%)	N (%)	N (%)	
**Age, years**	≤50	549 (59.9)	115 (72.8)	664 (61.8)	0.002
	>50	367 (40.1)	43 (27.2)	410 (38.2)	
**cT**	cT1	278 (30.3)	49 (31.0)	327 (30.4)	0.852
	cT2	638 (69.7)	109 (69.0)	747 (69.6)	
**Grading**	G1-2	218 (24.1)	42 (26.9)	260 (24.6)	0.481
	G3	685 (75.9)	114 (73.1)	799 (75.4)	
	missing	13	2	15	
**Histological**	NST	773 (84.4)	138 (87.3)	911 (84.8)	0.629
**type**	ILC	13 (1.4)	2 (1.3)	15 (1.4)	
	other	130 (14.2)	18 (11.4)	148 (13.8)	
**ypT**	ypT0/is	512 (60.0)	80 (55.2)	592 (59.3)	<0.001
	ypT1/2	341 (39.9)	60 (41.4)	401 (40.1)	
	ypT3/4	1 (0.1)	5 (3.4)	6 (0.6)	
	missing	62	13	75	
***BRCA1* or *BRCA2* mt**	no	404 (86.1)	60 (60.6)	464 (81.7)	<0.001
	yes	65 (13.9)	39 (39.4)	104 (18.3)	
	missing	447	59	506	
***BRCA1* mt**	no	416 (88.7)	62 (62.6)	478 (84.2)	<0.001
	yes	53 (11.3)	37 (37.4)	90 (15.8)	
	missing	447	59	506	
***BRCA2* mt**	no	457 (97.4)	97 (98.0)	554 (97.5)	1.000
	yes	12 (2.6)	2 (2.0)	14 (2.5)	
	missing	447	59	506	

BCS = Breast-conserving surgery; ILC = invasive lobular carcinoma; M = mastectomy; mt = mutated; NST = no special type.

Patterns of relapse are shown in Table 2. LRR (8.8% of patients) was the most common first site of recurrence, followed by distant relapse (7.2% of patients). Cumulative incidence of LRR at 5 years was significantly higher in patients with invasive residual disease in the breast (10.1% vs. 3.6%; p = 0.004; landmark analysis set) and for patients with increasing ypT-stage (ypT0/is 4.9% vs. ypT1 8.6% vs. ypT2/3 13.3%; p = 0.028 for ypT0/is vs. ypT1-3) and was numerically higher in patients who underwent mastectomy (12.31% vs. 6.71%; p = 0.136). Upon multivariate analysis, residual disease in the breast was the only factor that was significantly associated with LRR (HR 2.22 [95%- CI 1.38–3.58]; p = 0.002) (see Table 3).

**Table 2:** Patterns of recurrence.

Event	N	N in %
**Locoregional**	94	8.8
**Distant**	77	7.2
**Secondary malignancy**	21	2.0
**Death**	16	1.5

**Table 3:** Locoregional recurrence rates (LRR) and results of multivariate Cox-regression analysis.

Parameter	Category	5-year LRR (%)	Hazard ratio (95%CI)	*P*-value
**Surgery type**	BCS	6.71 (5.11–8.60)	1.213 (0.673–2.187)	0.5209
	mastectomy	12.31 (7.17–18.93)		
**Age**	≤50	7.59 (5.60–9.97)	1.090 (0.709–1.675)	0.6949
	>50	7.25 (4.85–10.27)		
**cT**	cT1	6.66 (4.14–9.98)	1.088 (0.622–1.902)	0.7676
	cT2	7.74 (5.85–9.97)		
**ypT (3 groups)**	ypT0/is	4.88 (3.24–7.01)	not included	n. a.
	ypT1	8.64 (5.68–12.36)		
	ypT2/3	13.34 (6.99–21.75)		
**pCR (landmark)**	no pCR	10.11 (7.57–13.06)	2.223 (1.381–3.578)	0.0010
	pCR	3.57 (2.12–5.61)		

BCS = Breast-conserving surgery; CI = confidence interval; n.a. = not analyzed; pCR = pathologic complete response.

**Fig. 2:**
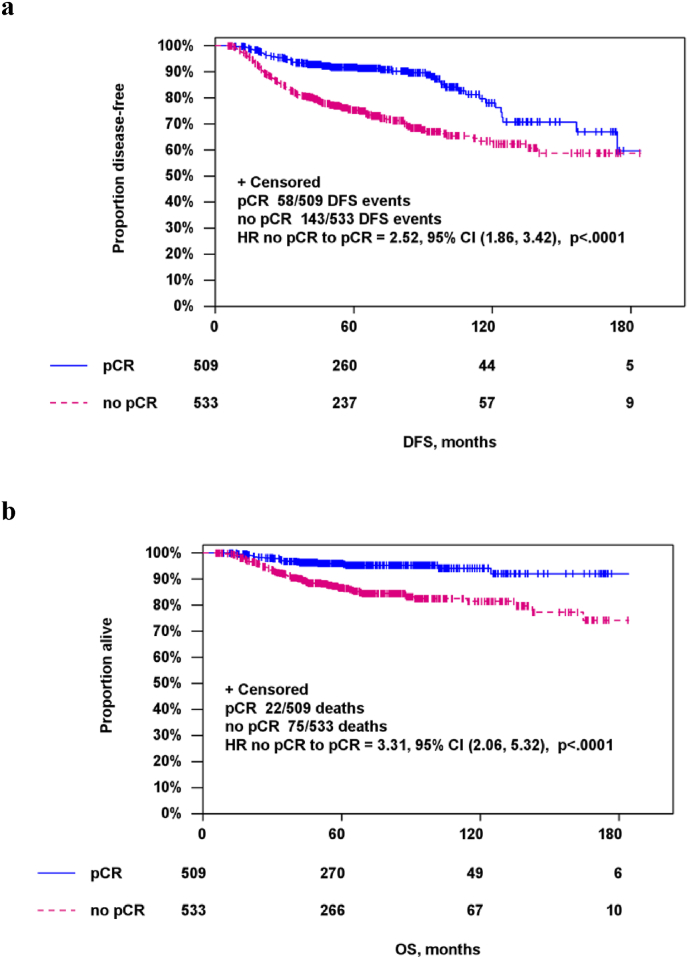
Disease-free survival (a, DFS) and Overall survival (b, OS) according to pathologic complete response (pCR).

DFS was significantly better in patients that underwent BCS (HR 0.47 [95%-CI 0.34–0.66]; p < 0.001]) while it was significantly impaired in patents with residual disease in the breast (HR 2.52 [95%-CI 1.86–3.42]; p < 0.001), Kaplan-Meier-curves are shown in Fig. 2. This was confirmed using multivariate Cox-regression analysis, results are shown in Table 4.

**Table 4:** Multivariate Cox-regression analysis for disease-free survival (DFS) and overall survival (OS).

		Multivariate Cox-regression analysis – DFS		Multivariate Cox-regression analysis – OS	
Parameter	Category	Hazard ratio (95%CI)	*P*-value	Hazard ratio (95%CI)	*P*-value
**Surgery type**	BCS	0.51 (0.36–0.72)	<0.001	0.43 (0.27–0.68)	<0.001
	mastectomy				
**Age**	≤50	1.03 (0.77–1.37)	0.855	1.01 (0.67–1.53)	0.948
	>50				
**cT**	cT1	0.71 (0.50–1.01)	0.058	0.76 (0.46–1.28)	0.306
	cT2				
**pCR (landmark)**	no pCR	2.43 (1.78–3.31)	<0.001	3.15 (1.94–5.10)	<0.001
	pCR				

BCS = Breast-conserving surgery; CI = confidence interval; pCR = pathologic complete response.

**Fig. 3:**
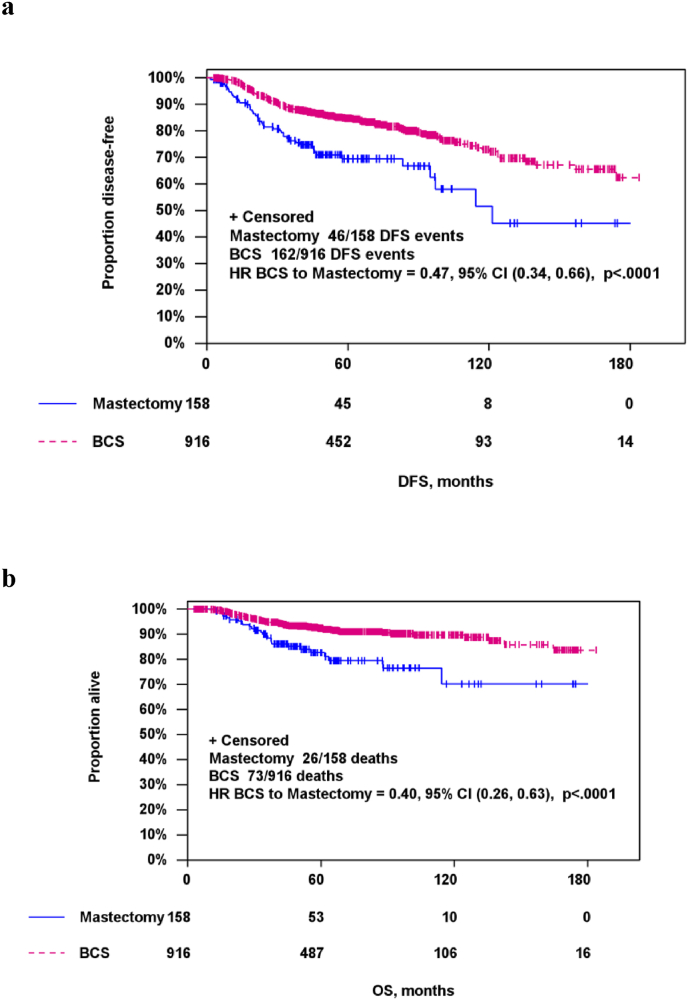
Disease-free survival (a, DFS) and Overall survival (b, OS) according to type of surgery (BCS = breast-conserving surgery ).

Similar results (Fig. 3) were found for OS (BCS vs. mastectomy HR 0.40 [95% CI 0.26–0.63]; p < 0.001; non-pCR vs. pCR HR 3.31 [95% CI 2.06–5.32]; p < 0.001). Again, multivariate analysis confirmed these findings (Table 4).

## 3 Discussion

Using data from a meta-database of NACT-trials in patients with early-stage node-negative TNBC, we demonstrate comparable locoregional control and improved DFS and OS for patients underwent BCS as compared to mastectomy. Patients that achieved a pCR had improved locoregional control, DFS and OS.

The finding of improved locoregional control and survival in patients with pCR is not surprising. The prognostic value of pCR regarding survival has been consistently demonstrated [11]. Furthermore, there is growing evidence that pCR is also strongly correlated with improved locoregional control [29,30].

The optimal locoregional management of TNBC has been a matter of debate for the past decades. A population-based analysis from Canada assessed the outcome of 768 patients with TNBC that underwent up-front surgery [9]. Mastectomy without radiotherapy was the only factor significantly associated with increased LRR (HR 2.53 [95% CI 1.12–5.75]). Upon multivariate analysis, there was no difference in OS (HR 1.31 [95% CI 0.81–1.92]). Even in matched patients with pT1-2 pN0 TNBC, LRR-free survival was inferior with mastectomy compared to BCT (p = 0.039).

A population-based study with a similar design reported the outcome of TNBC-patients using the Surveillance, Epidemiology, and End Results (SEER)-database [31]. The study included 12761 patients with T1-2 N0 TNBC and compared OS of patients who underwent BCT to those who received mastectomy alone after propensity-score matching. The results demonstrated significantly improved OS and breast cancer-specific survival (BCSS) for patients treated with BCT. These results were consistent regardless of the use of chemotherapy. Women older than 60 years had a worse overall prognosis and derived a significant benefit from BCT while the outcome for women under the age of 40 years and those aged 40–60 years was not affected by local therapy suggesting that older women may be undertreated with mastectomy.

It could be hypothesized that the improvements seen in the above-mentioned studies are due to the use of adjuvant radiotherapy. Regarding the OS benefit of adjuvant radiotherapy after BCS, Algan et al. analyzed the outcome of 44731 TNBC-patients from the National Cancer Database [32]. Radiotherapy after BCS was associated with improved OS for patients <70 years as well as those aged ≥70 years.

Kindts et al. studied an institutional database and identified 439 patients with TNBC [33]. They grouped patients according to local therapy. Upon multivariate analysis, LRR was comparable for BCT compared to mastectomy (HR 1.35 [95%-CI 0.56–3.25]) but further results were heterogenous. Even though BCSS was inferior with mastectomy (HR 1.84 [95%-CI 1.00–3.36]) overall, there were no significant differences in LRR and BCSS for patients with T1-2 N0 with negative margins. Patients treated with mastectomy and radiotherapy had superior locoregional control (HR 3.39 for BCT vs. mastectomy + RT [95%-CI 1.08–10.06]) but comparable BCSS (HR 1.56 [95%-CI 0.81–3.02]) compared to BCT-patients.

There are limited data regarding the optimal locoregional management of breast cancer explicitly after NACT. The previously mentioned EBCTCG meta-analysis that demonstrated increased LRR-rates has been used to argue against the use of NACT [25]. This publication showed increased rates of LRR both in patients with planned mastectomy before NACT as well as in patients who were downstaged from mastectomy to BCS. However, it is important to interpret these data in the context of improvements in diagnostic imaging, pathologic evaluation of residual disease and margin status as well as increased rates of pCR through improved systemic therapies. There is one retrospective analysis comparing BCT and mastectomy after NACT in a cohort of 1641 patients that demonstrated improved DFS, distant-metastasis free survival and OS with BCT [34]. This analysis was adjusted for multiple factors, including subtype, but no subgroup analyses were presented.

Mamtani et al. analyzed a cohort of 685 patients who were BCS-eligible after NACT [35]. Among these, 282 patients (41%) were initially BCS-ineligible and chose BCT while 160 patients (23%) were initially BCS-ineligible and chose mastectomy. The risk of LRR was not significantly increased for patients that chose BCT as compared to those who chose mastectomy or patients that were BCS-eligible before NACT (p = 0.17), although 58.8% of mastectomy-patients received adjuvant radiotherapy. Golshan et al. analyzed data from the BrighTNess-trial that evaluated the incorporation of Carboplatinum and Veliparib into the neoadjuvant treatment of patients with stage II-III TNBC [36]. More than half of patients who were initially planned for mastectomy converted to BCT-eligible. However, only 68.1% of the 342 patients that were deemed BCT-eligible underwent BCT with BCT-rates being significantly higher in Europe and Asia compared to North America (odds ratio, 2.66 [95% CI, 1.84–3.84]). This suggests that BCT may be underutilized with significant geographical variations.

Recently, a retrospective report from the I-SPY2 trial was published [37]. The authors analyzed the locoregional outcome of 1462 patients according to the type of surgery. There was no significant difference in terms of LRR between patients who underwent BCT vs. mastectomy. Treatment response was analyzed using the residual cancer burden-index and was highly associated with LRR. Type of surgery was not associated with distant recurrence-free survival or OS. The LRR-rate in the BCT (5.4%) and mastectomy-group (7.0%) was slightly lower than in our experience, however the association between treatment response and LRR is comparable to our findings. It is important to point out that the cohort from the I-SPY2 trial is much more heterogeneous in terms of initial (cT1-4 cN0/+) and pathological tumor stages (ypN0/+) and tumor biology (all subtypes allowed) than our analysis. This might explain the different findings in terms of distant recurrence-free survival or OS.

The main limitation of our work is its retrospective design. Although we tried to incorporate major prognostic factors, there still remains a relevant risk of uncontrolled confounding variables such as RT, lymphovascular invasion or multifocality/-centricity. As stated in the results part, patients undergoing BCS were significantly older and had a higher pCR rate which may have contributed to the better outcome in the BCS-group. This selection bias may explain the survival benefit, that would not be expected from BCS and adjuvant radiotherapy alone. Younger patients and patients with BRCA-mutation received mastectomy significantly more often, demonstrating further drivers of local therapy decisions which may also impact outcome. However, BRCA-mutation status could not be determined for 47% of patients. Data regarding adjuvant radiotherapy were not available. Therefore, causality cannot be inferred from our data. However, in a previous analysis of three prospective GBG-trials, 98.5% of patients received adjuvant RT after BCS [27,28]. We selected a subgroup of patients that had no recommendation for adjuvant post-mastectomy RT in the study guidelines. Nevertheless, a subset of patients may have received post-mastectomy RT, which would most likely affect the results in favor of the mastectomy-group. Similarly, omission of adjuvant RT after BCS could have negatively impacted the outcome in the BCS-group. Both major limitations should have influenced our results in favor of mastectomy. Still, our results concerning LRR and survival remain in favor of BCS. It seems to be a safe assumption, that there are no reasons to suspect that BCT in TNBC after NACT is inferior to mastectomy without RT. This insight is of high relevance for clinicians as younger patients receive radical, non-beneficial surgery more often, possibly driven by safety concerns [38].

## 4 Conclusions

BCS following neoadjuvant chemotherapy was not associated with an increased risk of LRR in patients with early stage TNBC. Use of BCS was associated with superior DFS and OS compared to mastectomy. Treatment response was the major determinant of favorable outcome. TNBC-subtype should not be used as an argument to withhold BCS followed by adjuvant RT in eligible patients.

## Funding

We acknowledge financial support by DFG within the funding programme Open Access Publikationskosten. The original trials were funded as described: GeparDuo was funded by Aventis, Amgen and Chugai; GeparTrio (pilot and main study) was funded by Aventis, Amgen and Roche; GeparQuattro was funded by Amgen, Roche and Sanofi-Aventis; GeparQuinto was funded by Celgene and Roche; GeparSixto was funded by GlaxoSmithKline, Roche and Teva; GeparSepto was funded by Celgene and Roche; and GeparOcto was funded by Amgen, Roche, Teva and Vifor.

## CRediT authorship contribution statement

**David Krug:** Writing – review & editing, Writing – original draft, Formal analysis, Conceptualization. **Valentina Vladimirova:** Writing – review & editing, Writing – original draft, Project administration, Formal analysis. **Michael Untch:** Writing – review & editing. **Thorsten Kühn:** Writing – review & editing. **Andreas Schneeweiss:** Writing – review & editing, Investigation, Funding acquisition. **Carsten Denkert:** Writing – review & editing. **Beyhan Ataseven:** Writing – review & editing, Conceptualization. **Christine Solbach:** Writing – review & editing. **Bernd Gerber:** Writing – review & editing, Conceptualization. **Hans Tesch:** Writing – review & editing. **Michael Golatta:** Writing – review & editing, Conceptualization. **Sabine Seiler:** Writing – review & editing, Project administration. **Jörg Heil:** Writing – review & editing, Conceptualization. **Valentina Nekljudova:** Writing – review & editing, Formal analysis, Data curation, Conceptualization. **Johannes Holtschmidt:** Writing – review & editing, Project administration, Formal analysis. **Sibylle Loibl:** Writing – review & editing, Resources, Funding acquisition, Formal analysis, Conceptualization.

## Declaration of competing interest

DK reports honoraria from Merck Sharp & Dome, med update, onkowissen, best practice onkologie, ESO, ESMO, Astra Zeneca and Pfizer, advisory boards for Gilead as well as research funding from Merck KGaA, all outside the submitted work.

MU declares honoraria from AstraZeneca, Art tempi, Amgen, Daiji Sankyo, Lilly, Roche, Pfizer, MSD Oncology, Pierre Fabre, Sanofi-Aventis, Myriad, Seagen, Gilead and Novartis; he also reports honoraria or fees for consulting or advisory role from Amgen, Lilly, Roche, Pfizer, Lilly, MSD, Pierre Fabre, Novartis, MSD Oncology, Roche, Agendia, Pierre Fabre, Seagen, Gilead, Lily, Stemline and Genzyme. All honoraria and fees paid to the employer/institution.

TK reports honoraria from MSD, Pfizer, Gilead, Astra Zeneca, Daiichi Sankyo, Roche, Merit Medical, Endomagnetics, Sirius Medical, Hologic.

AS declares grants from Celgene, Roche and AbbVie; personal fees from Celgene, Roche, Pfizer and AstraZeneca for travel expenses outside the submitted work. AS received honoraria from Roche, Celgene, Pfizer, AstraZeneca, Novartis, MSD, Tesaro, Lilly, Seagen, Gilead, GSK, Bayer, Amgen and Pierre Fabre outside the submitted work outside the submitted work.

BA reports honoraria for lectures from Roche, Astra Zeneca, GSK, MSD, Celgene, Lilly, Novartis, Eisai and advisory board for MSD, GSK, Amgen, Sanofi-Aventis, Eisai.

SS reports personal fees from Abbvie outside the submitted work. SS declares to be an employee of GBG Forschungs GmbH. GBG Forschungs GmbH received funding for research grants from Abbvie, Amgen, AstraZeneca, BMS, Daiichi-Sankyo, Gilead, Molecular Health, Novartis, Pfizer and Roche (paid to the institution); other (non-financial/medical writing) from Daiichi-Sankyo, Gilead, Novartis, Pfizer, Roche and Seagen (paid to the institution). GBG Forschungs GmbH has licensing fees from VMscope GmbH. In addition, GBG Forschungs GmbH has a patent EP21152186.9 pending, a patent EP19808852.8 pending, and a patent EP14153692.0 pending.

VN declares to be GBG Forschungs GmbH employee. GBG Forschungs GmbH received funding for research grants from Abbvie, Amgen, AstraZeneca, BMS, Daiichi-Sankyo, Gilead, Molecular Health, Novartis, Pfizer and Roche (paid to the institution); other (non-financial/medical writing) from Daiichi-Sankyo, Gilead, Novartis, Pfizer, Roche and Seagen (paid to the institution). GBG Forschungs GmbH has licensing fees from VMscope GmbH. In addition, GBG Forschungs GmbH has a patent EP21152186.9 pending, a patent EP19808852.8 pending, and a patent EP14153692.0 pending.

JH reports personal fees and non-financial support from Daiichi Sankyo, non-financial support from Hologic, personal fees from MSD Oncology, personal fees from Novartis, personal fees from Palleos Health Care, personal fees from Pfizer, personal fees from Roche Pharma, personal fees from Seagen, outside the submitted work. JH declares to be GBG Forschungs GmbH employee. GBG Forschungs GmbH received funding for research grants from Abbvie, Amgen, AstraZeneca, BMS, Daiichi-Sankyo, Gilead, Molecular Health, Novartis, Pfizer and Roche (paid to the institution); other (non-financial/medical writing) from Daiichi-Sankyo, Gilead, Novartis, Pfizer, Roche and Seagen (paid to the institution). GBG Forschungs GmbH has licensing fees from VMscope GmbH. In addition, GBG Forschungs GmbH has a patent EP21152186.9 pending, a patent EP19808852.8 pending, and a patent EP14153692.0 pending.

SL reports institutional COIs: The institute receives grants and other from Abbvie, other from Amgen, grants and other from AstraZeneca, other from BMS, grants and other from Celgene, grants, non-financial support and other from Daiichi-Sankyo, other from Eirgenix, other from Eisai Europe Ltd, other from GSK, grants, non-financial support and other from Immunomedics/Gilead, other from Lilly, other from Merck, grants from Molecular Health, grants, non-financial support and other from Novartis, grants, non-financial support and other from Pfizer, other from Pierre Fabre, other from Relay Therapeutics, grants, non-financial support and other from Roche, other from Sanofi, non-financial support and other from Seagen, other from Olema Pharmaceutics, other from VMscope GmbH, outside the submitted work; In addition, Dr. Loibl has a patent EP21152186.9 pending, a patent EP19808852.8 pending, and a patent EP14153692.0 pending.

All other authors report that they have no conflicts to declare.
